# Does Implantation of an Artificial Soft Anal Band Provide an Opportunity for Improvement of Biopsychosocial Function in Patients with Severe Fecal Incontinence?

**DOI:** 10.1155/2019/9843164

**Published:** 2019-10-29

**Authors:** Dorota Żelazny, Michał Romaniszyn, Piotr Wałęga

**Affiliations:** Third Chair of Surgery, Jagiellonian University Medical College, Krakow, Poland

## Abstract

**Introduction:**

Severe fecal incontinence describes a condition of complete loss of control over fundamental physiological functions and loss of abilities to fulfil psychosocial functions by the patients. The last-step procedure, that is, to restore hope for improvement of biopsychosocial functioning and quality of life determined by the patient's health status is implantation of an artificial anal sphincter.

**Objective:**

The study was a comparative analysis of the effect of the employed surgical procedure upon the degree of defecation control and quality of life indices in its behavioral, mental, and social aspects prior to and 3, 6, and 12 months postoperatively. The analysis also included the effect of the patient's individual style of coping with stress and the functional outcome of the procedure.

**Material and Methods:**

The study included a group of 12 patients: 6 females and 6 males, aged from 36 to 60 years of life. The tools consisted of scoring systems that measured symptom intensity (FISI and Jorge and Wexner scale). In assessing the psychosocial functioning, the authors employed the Fecal Incontinence Quality of Life Scale (by Rockwood). The individual mode of coping with the disease was evaluated by using the CISS scale by Endler and Parker.

**Conclusions:**

The analysis of results demonstrated that the procedure of implanting an artificial anal sphincter affected the “continence” (up to 50–60% postoperatively) and led to improvement in psychosocial functioning in all its assessed aspects, i.e., lifestyle, employment of precautionary measures, depression, anxiety, and embarrassment. It was also noted that due to the specific character of the procedure (the necessity to operate an artificial implant), better mean results in assessment of the procedure functionality were achieved by patients presenting the goal-concentrated mode rather than emotions-concentrated mode of coping with the disease. Thus, it seems justified to state that assessment of biopsychological functioning may be a good criterion of the procedure effectiveness.

## 1. Introduction

Severe fecal incontinence describes a condition of a complete loss of control over bowel movements where the method of choice is the last-step procedure—an advanced reconstruction or abdominal stomy. These procedures trigger a tremendous emotional strain and imply a conflict between the needs and expectations and the anticipated anxiety associated with the surgical procedure itself.

In spite of the above, patients with severe fetal incontinence resulting from damage to the anal sphincter apparatus seek help in the field of reconstructive surgery in view of the devastating effect of loss of control over basic physiological functions upon their entire sphere of psychosocial functioning. It results in a drastic decrease in quality and prosperity of life that are determined by the patient's state of health.

We do not know which element of the patient's psyche prompts him to reach a decision to be subjected to an extensive and strenuous surgical procedure and in what way it affects the improvement of the aforementioned “quality of life” as a functional effect of the disease and its therapeutic management [1]. The last-step procedure that is capable of restoring the state approximating a definable model of health (which in the holistic-functional paradigm in force is treated as a mutual relation between all the functional structures of the human being and his environment) is transplantation of a hydraulic prosthesis—artificial anal sphincter (AAS).

AAS is a soft silicone band that fulfils the role of a mechanical valve. It is employed in patients with congenital (ankyloproctia) or acquired defects of the barrier mechanism (traumatic injuries of the pelvis or spinal cord and systemic diseases) in whom, due to lack of anal sphincter or its degradation, conservative treatment or other surgical procedures cannot be possibly performed. Such operations, successfully done in Europe and the United States, were carried out for the first time in Poland in the center employing the authors in the period 2001–2007.

## 2. Objective

The objective of the study was the analysis of the somato-mental state of patients qualified for the procedure of AAS implantation and in what way the implantation of an artificial anal sphincter changed the selected indices of quality of life in the behavioral, mental, and social aspects of patients' functioning. Of significance was also determining the correlation between the objectivizing symptomatic examinations and the subjective sense of controlling one's body. The authors also took under consideration whether individual traits of the patient, such as coping with stress, affected the improvement of the aforementioned aspects of quality of life and whether assessment of psychosocial functioning of the patients might provide a good criterion of effectiveness of the procedure.

## 3. Methods

In order to objectivize the assessment of the degree of bowel control, the authors employed questionnaires that measured the intensity of symptoms, i.e., the Jorge–Wexner scale [2] which describes the type of stool, frequency, and the necessity to change lifestyle (including wearing sanitary pads) on a scale 0 to 20, where 0 means complete control of stool and gas and 20 means total fecal incontinence. The second of the scales used is FISI(the Fecal Incontinence Scoring System) [3] which determines the frequency of incontinence of solid and liquid stool and gas and mucus, on a scale 0 to 61, where 0 describes total lack of incontinence and 61 describes no control of physiological functions. The quality of psychosocial functioning was evaluated using the Rockwood scale—Fecal Incontinence Quality of Life, composed of a total of 29 items. These items form four scales: Lifestyle (10 items), Coping/Behaviour (9 items), Depression/Self-Perception (7 items), and Embarrassment (3 items), analyzing dynamic relations between general condition, physiological functions, and their influence on emotional, behavioral, and social zone (with the scores from 0 to 4 and in depression scale from 0 to 6, where a higher score means better coping with depression) [3]. The evaluation was carried out prior to the procedure of implanting the artificial anal sphincter (AAS) and 3, 6, and 12 months postoperatively. In order to determine the style of coping with stress (focused on the task, emotions, or avoidance), the patients were also tested once, before the surgery, with a CISS scale by N. S. Endler and J. D. Parker [4]. The authors analyzed whether individual personality traits of the patient affected the evaluation of his functioning in the aspect of quality of life, as well as the implemented decision on being subjected to the procedure.

The questionnaire studies, performed both before and after the surgical procedure, were supplemented by extended, structurized clinical medical history-taking, where the patients responded to questions referring to the specific character of the procedure itself whether the implantation of the artificial device, the necessity of learning how to operate the implant, anxiety concerning the faultless functioning of the hydraulic band, or its rejection by the patient's organism as a foreign body affected their life activities, physical, mental, and social conditions. The diagnostic conversation also included an element consisting in psychoeducating the patient in making his expectations more realistic and getting adjusted to living with an artificial sphincter.

At each stage of the postoperative follow-up examinations, the authors also assessed individual satisfaction levels of the patients. The subjects responded to the question whether they did not regret having been subjected to the procedure of AAS implantation.

It should be mentioned that all the patients gave their consent to participate in the study and were informed they might refuse to answer certain questions at each stage of the investigation (numerous questions, both formulated by the authors and included in the questionnaires, addressed intimate areas) or withdraw their consent to further participate in the study. The Jagiellonian University Bioethical Committee granted their consent to the study.

## 4. Technique of Surgery

We used a special prosthesis designed basic on gastric band used in the treatment of morbid obesity-Soft Anal Band System (AAS, Agency for Medical Innovation; AMI Feldkirch Austria, CE Body number 0298, ID 170530317; Reg. No. 066924MR2). The prosthesis itself consists of three parts: elastic band, pressure regulating balloon (pump), filled intraoperatively with a radio contrast, and a valve used to regulate the pressure in the anal band. We used osmotic cleaning to prepare the colon before the procedure and metronidazole, an antibiotic, was given before (with premedication) and after surgery up to 7 days. The procedure was performed under general anesthesia, with the patient in lithotomic position. In the first step, perianal incisions are made and a tunnel is created around the anus. Specially designed flexible ruler helps to choose an appropriate cuff size. Anal band is then placed around the anus. During the next step, an incision is made over the pubis and the calibration's port is placed in the subcutaneous space anterior to the bladder. The pump is placed subcutaneously on the side on the patient's dominant hand. All components were filled with radio-opaque fluid and connected to create closed hydraulic system. Before discharge from the hospital, all the patients were repeatedly trained how to operate the system. The system was activated under manometric control proceeded by X-ray abdomen imaging, 4–6 weeks after procedure.

## 5. Results

The study was carried out in the third chair of surgery, Jagiellonian University Medical College, Krakow, Poland, in the period from 2001 to 2007. A group of 12 patients were implanted with artificial anal sphincter (none of the patients agreed to the proposal before colostomy procedure), 6 females and 6 males in the age range of 36 to 60 years of life.

The mean age of the patients was 45 years. Clinical trials were carried out before and 3, 6, and 12 months after surgery (except scale CISS, examination before surgery).

The quantitative characterization of the individuals qualified for the procedure with respect to the pathogenetic mechanism is given in Table 1.

In the follow-up period, patients remained under continuous surgical and psychological care in our Colorectal Ambulatory Center. In the follow-up, we observed, in one case, one major complication requiring explanation of the whole system: iatrogenic injury to the activator followed by infection of this site. In two other cases, the system was recalibrated, adding more fluid (after 3 months). In one case in our group, we observed minor wound dehiscence (2nd week) and one case requires additional sutures (2nd week). In the follow-up, both wounds healed without further complications. It should be mentioned that, in the preoperative period, in order to improve the consistency of stool, patients used loperamide, after surgery, occasionaly. The scores are obtained in the N. S. Endler and J. D. Parker questionnaire, in which the authors categorize coping styles employed in stressful situations into three principal modes, two of them being concentration on the task and concentration on emotions, with the third on avoidance, the latter being additionally divided into two subtypes, i.e., avoidance by engaging in alternative activities or in social life, presented in Table 2.

In studies using symptomatic scales JW and FISI, we observed significant improvement (in relation to the preoperative period) in ability to control defecation in the whole group (see Table 3).

**Figure 1:**
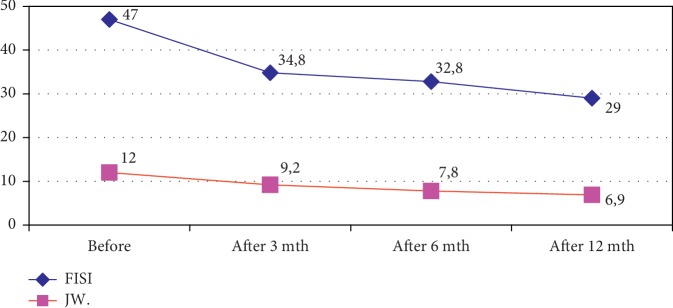
Assessment of the degree of fecal incontinence in the FISI and Jorge–Wexner scales before and 3, 6, and 12 months postoperatively.

The comparison of the scores of symptomatic scales achieved prior to and after the procedure of hydraulic prosthesis implantation is presented in Figure 1.

The results of patients presenting a specific style of coping with stress (disease-fecal incontinence) in FISI and JW scales are presented in Tables 4 and 5.

General results of symptomatic scales show significant improvement especially in the first three months after the operation, when the degree of “fecal continence” according to the FISI scale improved by 12.2 points, and in the JW scale by 2.8 points, improving slightly after 6 months (according to FISI, further improvement by 2 points and in JW, by 1.4 points) and after 12 months postoperatively (FISI-3.8 points and JW-0.9 points). The results presented in individual groups show a similar tendency to achieve continence.

In the FIQL scale that measures four aspects of psychosocial functioning, the mean scores were as given Table 6.

**Figure 2:**
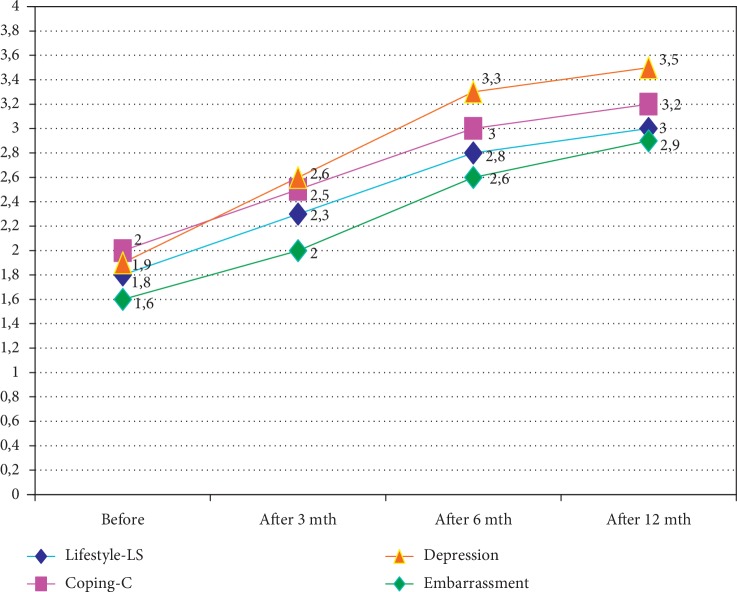
Quality of life assessment (FIQL scale) in all categories prior to and 3, 6, and 12 months postoperatively.

The assessment of all the four aspects of quality of life (FIQL) prior to the procedure and 3, 6, and 12 months postoperatively is illustrated in Figure 2.

Results in individual FIQL subscales (Lifestyle, Coping, Depression, Embarrassment) for each type of stress management are illustrated in Tables 7–10.

In the follow-up examination in the Lifestyle subscale, we observed an improvement by 0,5 points after 3 months and further increased by another 0,5 points after 6 months postoperatively. Twelve months after surgery, a low increase of the point scale (by 0,2 points) was still noticeable. In the Coping category, we observed an increase by 0,5 points after 3 and 6 months and by 0,2 points after 12 months after surgery. The authors noted a measurable improvement in the sphere of Embarrassment in the follow-up after 3 months—2.0 points (an increase by 0.4 point as compared to the initial value of 1.6 points), subsequently 2.2 points (an improvement by another 0.2 points after 6 months), and 2.5 points (an increase by 2.3 points after 12 months postoperatively). The relatively best result in pre- and postoperative evaluation was achieved in the sphere of coping with depression (Depression)—an increase by 0.7 point after 3 months as compared to the initial score of 1.9 points and by another 0.7 points after 6 months, what summarily amounted to 3.4 points, and by 0.2 points after 12 months postoperatively, finally yielding 3.6 points. Taking into consideration the individual results of the patients with different styles of coping with stress, it was noticed that the best satisfaction (compared to the initial scoring) in all subscales was presented by the task-concentrated group and then patients presenting a style of avoidance. The group concentrated on emotions presented the lowest satisfaction with the functional effect of the treatment.

## 6. Presentation of Results and Discussion

The review of medical literature demonstrates data on the employed method of implanting the hydraulic sphincter prosthesis, including the mode of implantation, possible postoperative complications (wound infection, necessity of employing additional sutures, and revision of the anal system, including explantation of the implant), and functional effects of the procedure itself understood as results of functional examinations and intensification of incontinence symptoms (Wang and Wexner). In the present study, the preparation of the patient, surgical technique, and outcomes did not differ from those described by other authors.

Nevertheless, one issue is still under question, namely, in what way the proposed and performed reconstruction procedure changes the functioning of the patient in its behavioral, cognitive, emotional, and social aspects. Such an assessment was performed only by Lehur et al. and was based on studies of the above authors employing the same investigative tools and investigating the implantation of the device in 19 patients in the years 1996–2000 [5] and by Carmona who studied quality of life after the procedure of implanting a hydraulic band [6].

Our results, similar tothe findings of Lehur and Carmona, show that the employed surgical technique markedly affected the sense of improved bowel control. This is illustrated in the patient's self-assessment based on the FISI and JW questionnaires. What seems significant is the answer to the question whether the fact that one has had an artificial anal sphincter implanted (and by the same token, a chance for regaining self-control over basic physiological functions) may change the remaining spheres of the patient's functioning. In other words, to be blunt, the question whether it may, and if so, in what way, change their lifestyle and reduce the level of anxiety and depression.

In the investigations carried out by the present authors, similar to the reports by Lehur and Carmona, a moderate increase of satisfaction was achieved in all the evaluated aspects of psychosocial functioning. Of particular importance was the significant decrease in depression level, which the presently investigated patients valued the most.

And thus, one might say that the postoperative sense of regaining self-control over defecation affected the modification of behavioral actions in the lifestyle sphere. In keeping with the patient's reports, this mostly resulted from the fact they no longer needed to use precautionary measures (pampers) to leave the safe zone, i.e., their home.

In the period up until the time of calibration and activation of the system, i.e., 4–6 weeks postoperatively, only six subjects used sanitary napkins as a precaution. After the activation of the band, two patients (in whom the system needed to be recalibrated in the later period) occasionally used panty liners, while the remaining subjects from the group could give them up. In spite of the necessity of caring for and treating the surgical wound, transient pain associated with the operation itself and the necessity to calibrate the band, as well as learning to operate the activator, the patients demonstrated favorable opinions with respect to the functional effects of the procedure.

As related by the patients, of special importance was freeing themselves from the necessity of staying close to a toilet and from strict control of nutritional habits. The effect was a higher activity in fulfilling their social needs, familial, societal and professional. For the patients, it meant possibilities of leaving their homes, going to the cinema or theater, staying outside their domicile at night, meeting their friends, and most of all undertaking professional activities. In association with increased mobility, three subjects changed their jobs for more interesting and more financially advantageous positions.

In case of six patients, the implantation of the artificial anal sphincter denoted undertaking sexual activity and a significant improvement in intimate relations with their partners since it allowed them to conquer their fear of discrediting themselves.

Twelve months after the surgery, the authors observed a slight increase of satisfaction in the lifestyle sphere, what denotes the effect of “establishing and getting used” to the new situation.

An implication of changes in the sphere of behavioral, and by the same token, also social functioning of the subjects, was a modification of the so-called cognitive schemas of the patients that were evaluated in the Coping category in the FIQL scale, that means beneficial change in self-perception, increased self-assessment, and in consequence regaining the sense of the value of one's body. Ten subjects declared that the image of “their own body with an implanted system, albeit alien but functioning and providing a sense of safety, was better than sanitary pads, adult diapers, and consequences of their using.” This exerted an effect on abandoning regressive defensive coping mechanisms characterized by avoidance and isolation, staying at home, in favor of mechanisms aiming at actions and higher activity levels, which was particularly apparent 12 months after the operation.

That corresponded to the degree of coping with depression, anxiety, and embarrassment that are prerequisites of stable emotional functioning. Thus, the predictability of bowel control reduces the episodes of anxiety and embarrassment in social situations, which was a secondary motivation for the patients to undertake more psychosocial activities.

The period between the operation and the first follow-up examination is the period of uncertainty associated with adaptation to and acceptance of the implanted system and its functioning. It is the period of the necessary learning to operate the system, but also of anxiety of the inability to defecate when the band fails to loosen up. Nevertheless, the results of the evaluation indicate an improvement in all the spheres of quality of life. It should be added that anxiety regarding the quality of the band functioning involved solely the first follow-up period after the operation.

Similarly, as in the case of the abovementioned studies, the present results after 6 and 12 months confirm further reduction of the level of anxiety and uncertainty, which is an effect of acceptable control and time-associated predictability of one's physiological functions.

The relatively best results in pre- and postoperative evaluation were achieved in the sphere of coping with depression (Depression). It proves that the ability to cope with sadness, regrets, negative thoughts involving oneself, and one's future provides a distance from their cause. Eighty percent of the subjects reported that the previously experienced sense of hopelessness and lack of satisfaction over their lives associated with their inability of controlling basic physiological needs did decrease.

As it follows from the studies of Lehur, improvement in evaluation of quality of life is the most significant within the medial interval after the surgery (approximately 3 to 6 months), and subsequently, it improves only slightly and may even show decreasing tendencies [5]. In the present study, the increase of satisfaction after the procedure was the highest in the first two monitored time intervals, after 3 and 6 months, while 12 months after the surgical intervention, it improved only slightly, which results from the mechanism of habituation.

The satisfaction of the patients was also evaluated in view of their personal traits (which was a brand-new aspect in this study): the style of coping in difficult situations (most assuredly, such a situation is represented by experiencing the disease, surgical procedure, and its consequence of being dependent on an artificial implant) using the CISS scale. When comparing the scores obtained using the above scale with individual data from the quality of psychosocial functioning scale, the authors noted that in a similar situation, implantation of an artificial anal sphincter, the best coping results and the highest level of satisfaction were characteristic of patients presenting the defensive style, aiming at actions and activity and searching for solutions of the problem, and somewhat poorer results were achieved by subjects who avoided problems using various denial methods or who searched for alternative solutions The lowest degree of satisfaction derived from the effect of the operation was expressed by the subjects presenting the style based on emotions. It can be said that personality traits naturally cannot be the main determining factor that qualifies to the implantation of AAS; however, they certainly may affect the functional effect of the surgery (in the patient's individual evaluation). It seems interesting to answer the question whether the “fight with your own fecal incontinence” undergoing surgery, may have an impact on changing the way of coping with stress. Such research would be very interesting from a psychological point of view.

On the other hand, the individual comparative score addressing the benefits derived from the procedure (data from the medical history-taking) with respect to the mechanism of sphincter damage pathogenesis indicated that the patients who suffered from ankyloproctia or anal sphincter defects since birth perceived the effect of band implantation as more beneficial since they lacked the previous experience of any type of control over the act of defecation and did not go through the trauma of losing such control.

Of significance is also the fact that there was no confirmation of the signaled preoperatively anxiety of the possibility of rejecting a foreign object by the patient's body and difficulties in ability to operate the implant. The fact that expectations concerning the functional effect of the procedure itself and also some limitations associated with the functioning of the entire system earlier rendered more realistic seemed to trigger beneficial results in adaptation to transient difficulties related to the after-effects of the very procedure.

In spite of the fact that preparation for surgery was associated with a number of unpleasant examinations, oftentimes of an intimate character, the subjects experienced perioperative anxiety and stress as well as uncertainty associated with their ability of coping with a new situation following AAS implantation and obligation to participate in the follow-up program; all the patients declared they did not regret having undergone the procedure of hydraulic band implantation (including 1 individual in whom the system had to be explanted). The authors even observed a tendency towards a better opinion on the benefits of the procedure as compared to the data from the questionnaires that objectivized the degree of bowel control (the FISI and Jorge–Wexner scales). The phenomenon, also described by other authors as the psychological continence or the mechanism of “mental stool retention”, to a high degree, affects all the evaluated spheres of life. It may suggest that preoperative degradation of quality of life and mental costs associated with the surgery itself as well as the chance for any kind of control exerts a motivating effect on the patient's self-perception as a “continent” person. One might then agree with Carmona et al. that, in case of an appropriate selection of patients, appropriate preparation of a multidisciplinary therapeutic team, in spite of possible postoperative complications, retaining the soft anal sphincter band significantly improves the overall biopsychosocial functioning of the patient in its quality of life aspect.

## 7. Conclusions

1. The results of the present comparative analysis of the effect of the performed surgical procedure, namely, implantation of an artificial anal sphincter show that it significantly affects the control over the act of defecation.
2. The possibility of controlling and anticipating bowel movements implies changes in all the domains of psychosocial functioning.
3. Appropriate preparation, psychoeducation, and personality traits of the patients affect the surgical effect.
4. Investigating the postoperative psychosocial functioning of the patients may be a good criterion of the procedure effectiveness.

Despite the risk of failure and possible complications, the procedure of implanting an artificial anal sphincter is an operation that is acceptable for the patients with severe fecal incontinence, and it significantly improves their quality of life.

Of utmost significance is regression of the anxiety and depression levels which allows for returning to acceptable forms of everyday social life.

Evaluation of particular spheres of quality of life in the process of qualification for surgery is important in achieving a “good functional-psychological response”, also in the sense of psychological continence.

## Figures and Tables

**Table 1:** Quantitative division of patients according to pathogenetic mechanism of anal sphincter damage.

Mechanism of pathogenesis	Congenital defects ankyloproctia	Spinal injuries with denervation syndrome	Extensive mechanical pelvic injuries	Hemorrhoidectomy
Subjects	3	3	2	4

**Table 2:** Quantitative division of patients according to style of coping with stress.

Style concentrated on:	Task	Emotions	Avoidance by engaging in alternative activities	Avoidance by engaging in social life
Subjects	4	4	2	2

**Table 3:** The average of the results obtained in FISI and JW scales.

Evaluation time	0	3	6	12
FISI scale	47,0	34,8	32,8	29,0
JW scale	12	9,2	7,8	6,9

**Table 4:** Scale FISI, results obtained due to the type of stress management.

	0	3	6	12
Group—style concentrated on task	47	34,8	32,5	30,6
Group—style concentrated on emotions	46,0	32,6	30,8	28,0
Group—style concentrated on avoidance, engaging in alternative activities	46,5	32,2	31,8	28,8
Group—style concentrated on avoidance, engaging in social life	47,0	34,6	33,8	29,2

**Table 5:** Scale JW, results obtained due to the type of stress management.

	0	3	6	12
Group—style concentrated on task	12,6	9,6	8,0	7,0
Group—style concentrated on emotions	11,6	9,0	7,4	6,5
Group—style concentrated on avoidance, engaging in alternative activities	12,0	9,2	7,8	7,0
Group—style concentrated on avoidance, engaging in social life	14,8	9,0	7,8	7,4

**Table 6:** The assessment of all the four aspects of quality of life (FIQL) prior to the procedure and 3, 6, and 12 months postoperatively.

	0	3	6	12
Lifestyle	1,8	2,3	2,8	3,0
Coping	2,0	2,5	3,0	3,2
Depression	1,9	2,6	3,3	3,5
Embarrassment	1,6	2,0	2,6	2,9

**Table 7:** Results of patients presenting different styles of coping with stress on the Lifestyle subscale.

	0	3	6	12
Group—style concentrated on task	1,4	2,3	3,0	3,2
Group—style concentrated on emotions	1,6	2,0	2,4	2,5
Group—style concentrated on avoidance, engaging in alternative activities	2,0	2,2	2,6	2,9
Group—style concentrated on avoidance, engaging in social life	2,2	2,5	2,9	3,2

**Table 8:** Results of patients presenting different styles of coping with stress on the Coping subscale.

	0	3	6	12
Group—style concentrated on task	1,8	2,6	3,2	3,4
Group—style concentrated on emotions	2,0	2,2	3,0	3,0
Group—style concentrated on avoidance, engaging in alternative activities	1,8	2,2	2,8	3,0
Group—style concentrated on avoidance, engaging in social life	2,2	2,5	3,0	3,2

**Table 9:** Results of patients presenting different styles of coping with stress on the Depression subscale.

	0	3	6	12
Group—style concentrated on task	1,8	2,8	3,4	3,9
Group—style concentrated on emotions	2,0	2,4	3,1	3,3
Group—style concentrated on avoidance, engaging in alternative activities	2,1	2,4	3,4	3,5
Group—style concentrated on avoidance, engaging in social life	2,0	2,0	3,0	3,2

**Table 10:** Results of patients presenting different styles of coping with stress on the Embarrassment subscale.

	0	3	6	12
Group—style concentrated on task	1,4	2,1	2,6	3,4
Group—style concentrated on emotions	1,6	1,8	2,4	3,0
Group—style concentrated on avoidance, engaging in alternative activities	1,8	2,0	2,4	2,7
Group—style concentrated on avoidance, engaging in social life	1,6	1,9	2,3	2,6

## Data Availability

The data will be available upon request to the corresponding author (dorota.ze@op.pl).
